# Stigma by association and family burden among family members of people with mental illness: the mediating role of coping

**DOI:** 10.1007/s00127-016-1256-x

**Published:** 2016-06-29

**Authors:** Remko L. M. van der Sanden, John B. Pryor, Sarah E. Stutterheim, Gerjo Kok, Arjan E. R. Bos

**Affiliations:** 1 Department of Work and Social Psychology, Faculty of Psychology and Neuroscience, Maastricht University, PO Box 616, 6200 MD Maastricht, The Netherlands; 2 Department of Psychology, Illinois State University, Normal, USA; 3 Faculty of Psychology and Educational Sciences, Open University, Heerlen, The Netherlands

**Keywords:** Stigma by association, Family burden, Psychological distress, Quality-of-life, Coping, Mental illness

## Abstract

**Purpose:**

When someone has a mental illness, family members may share the experience of stigma. Past research has established that family members’ experiences of stigma by association predict psychological distress and lower quality-of-life.

**Methods:**

The present study, conducted with 503 family members of people with mental illness examined the prevalence of 14 different coping strategies. Of greater importance, we examined the role of these coping strategies as mediators of the relationships between stigma by association and family burden, on the one hand, and outcomes, such as psychological distress and quality-of-life, on the other.

**Results:**

The results showed that both perceived stigma by association and family burden are associated with greater psychological distress and lower quality-of-life, and that most coping strategies mediate these relationships.

**Conclusions:**

Adaptive coping strategies were related to reduced negative outcomes, while most maladaptive coping strategies were related to enhanced negative outcomes. Implications for intervention development are discussed.

## Introduction

A stigma is a form of negative deviance that blemishes the identity and reputation of the person who bears the mark. It brands the bearer as someone to be avoided or socially excluded [11]. Research suggests that people with mental illness (PWMI) are stigmatized more severely than those with the other health conditions [8]. Research has also shown that people associated with individuals with mental illness can be stigmatized as well simply, because they are, in some way, connected to someone with a stigmatized identity [2, 38]. This phenomenon is called courtesy stigma or stigma by association (SBA) [2, 17]. In addition, family members of PWMI may also experience a range of practical struggles that constitute family burden. This can include financial problems, worries about the patient, time-consuming activities, missed career opportunities, and family quarrels [14, 20, 43].

Research has further shown that SBA and family burden can be major sources of psychological distress and diminished quality-of-life of family members of PWMI [26, 40, 46]. Psychological distress represents the negative mental health state recognisable by symptoms, such as anxiety, depression, negative affect, and loss of behavioural and emotional control [47], whereas quality-of-life represents one’s perception of one’s position in life within the systems and community, in which one lives, but also in relation to one’s expectations, standards, and goals [42]. SBA is also known to affect how family members view their family members with mental illness, and thus can negatively impact their interpersonal relationship and perceived closeness [9, 23]. As such, SBA and family burden not only have a strong and long-lasting effect on the quality-of-life of family members, but also on the well-being of their family members with mental illness [32, 45].

In seeking to mitigate the negative impact of SBA and family burden, family members use various coping strategies [25]. Lazarus and Folkman [25] defined coping as “constantly changing cognitive and behavioural efforts to manage specific external and internal demands that are appraised as taxing or exceeding the resources of the person”. Major and O’Brien [28] explored coping in specific relation to stigmatization, contending that stigmatization increases one’s exposure to potentially stressful situations. In fact, they claim that when stigmatization threatens one’s social status or identity, coping responses are triggered in an effort to regulate behaviour, cognitions, emotions, and the environment. In this process, people appear to appraise, first, the demands posed by the stigma and its potentially (negative) impact on well-being (primary appraisal), and, second, their resources and capabilities to cope with those demands (secondary appraisal; [25, 28]). These appraisals are important, and they have been found to predict the kind of coping strategy employed in stressful situations, such as stigmatization [25]. According to Lazarus and Folkman [25], if people believe they can manage or change the situation or condition, they are more likely to choose coping strategies geared to changing the stressor. If, however, they believe the situation or condition cannot be managed or changed, their coping efforts are more likely to be geared toward the regulation of emotions.

Coping strategies can also be viewed in terms of the degree to which they are adaptive [49]. In this context, adaptation refers to the degree to which one, when confronted with a stressor, successfully copes socially, physiologically, and psychologically [4]. Differences between the effects of coping strategies may, therefore, lie in the adaptiveness of coping strategies [21, 49]. Coping strategies, such as active coping, using emotional support, using instrumental support, planning, positive reframing, acceptance, and use of humour are considered adaptive, as they reduce stress levels and improve one’s functioning and quality-of-life . In contrast, coping strategies, such as self-distraction, denial, substance use, behavioural disengagement, turning to religion, venting negative emotions, and self-blame, are considered maladaptive, because they only temporarily mitigate the negative impact of the stressor and can even serve to amplify the stressful situation or condition. They are, therefore, considered counterproductive and ineffective in the long run [22, 30].

In-depth knowledge about SBA, family burden, the negative impact of both, and the coping strategies that can be employed to mitigate these negative effects is important in the context of developing effective intervention techniques among family members of PWMI. For this reason, the present study investigated the processes and mechanisms, by which these mitigating effects are produced. More specifically, we looked at the mediational effects of seven adaptive and seven maladaptive coping strategies on the associations between SBA and family burden, on the one hand, and psychological distress and quality-of-life , on the other, an approach that is, to our knowledge, relatively unique. Mediation analyses were considered most appropriate based on both the current literature (e.g., [7, 9, 13]), and on our previous qualitative findings [46] which indicated that we could expect to find a strong relationship between SBA and family burden, on the one hand, and psychological distress and quality-of-life, on the other hand. According to Holmbeck [19], mediation analyses are best conducted when strong relationships between the independent variable and the dependent variable are present.

We hypothesised (1) that SBA and family burden among family members of PWMI would independently predict increased levels of psychological distress and diminished quality-of-life; (2) that coping strategies would mediate the effects of SBA and family burden, on the one hand, and psychological distress and quality-of-life, on the other hand; (3) that the adaptive coping strategies would mitigate the effects of SBA and family burden on psychological distress and quality-of-life; and (4) that maladaptive coping strategies would exacerbate the effects of SBA and family burden on psychological distress and quality-of-life.

## Method

### Participants and procedure

In October 2013, immediate family members of PWMI in the Netherlands were recruited from an online panel (*N* = 14,170). This panel consisted of 4863 men (34.3 %) and 9307 women (65.7 %), with ages ranging from 12 to 85 years. In terms of level of educational attainment, 18.7 % of the panel had a low (i.e., elementary school or lower vocational training), 38.2 % medium (i.e., secondary or mid-level vocational training), and 43.1 % high (i.e., college or university) level of educational attainment. For the purposes of this study, panel members were first asked by e-mail whether they had a family member with mental illness, and if they would be willing to participate in a self-report study on being a family member of someone with mental illness. A positive response was given by 6840 panel members, and a random sample of 625 cases was, subsequently, drawn from these panel members. These panel members were then invited by e-mail to participate in the survey, and a reminder was sent 4 days after the initial invitation. Of those 625 cases, 503 panel members (i.e., 212 men and 291 women, aged 18–85 years (M 45.4, SD 13.4) completed the survey, yielding a response rate of 80.3 %. Informed consent was obtained, and participants were given points that could be exchanged for discount coupons upon survey completion. Participants’ demographic and background characteristics are displayed in Table 1.

**Table 1:** Demographic and background characteristics of sample (*N* = 503)

Variable	Percentage (%)
Family relationship	
Spouse	21.5
Child	21.4
Parent	34.4
Sibling	22.7
Gender	
Male	42.1
Female	57.9
Level of education^a^	
Low	22.1
Moderate	39.8
High	38.1
Marital status	
Single	17.5
Married	71.8
Divorced	9.3
Widowed	1.4
Ethnicity	
Dutch	97.2
Other	2.8
The type of mental illnesses experienced by participants’ family members^b^	
Depression	36.8
ADHD/ADD	21.2
Autism	19.2
Anxiety disorder	15.8
Bipolar disorder	11.0
Personality disorder	9.0
Schizophrenia or psychotic disorder	6.8
Other	10.3

^a^Low = elementary school or lower vocational training; moderate = secondary school or mid-level vocational training; high = college or university

^b^Because participants were allowed to select more than one mental disorder, the sum of the percentages exceeds 100 %

The study was approved by the Ethics Committee at Maastricht University’s Faculty of Psychology and Neuroscience.

### Measures

Stigma by association was assessed using a 28-item SBA Scale [44] that measures participants’ cognitive, emotional, and behavioural reactions to being related to someone with a stigmatized condition. Items (e.g., “People may treat me negatively if they find out that I have a family member with mental illness”, “When the person with the mental illness and I are in public, I pretend that we are not related”) were rated on a five-point scale ranging from strongly disagree (1) to strongly agree (5). A higher score indicates greater SBA. Cronbach’s alpha was .88.

Family burden was measured with a seven-item Burden Scale [38, 37]. Items (e.g.,: “It caused financial hardships in our family”, “It is time consuming having a family member with a mental illness”) were rated on a five-point scale ranging from strongly disagree (1) to strongly agree (5). A higher score is considered indicative of greater family burden. Cronbach’s alpha was .71.

Psychological distress was measured using the 18-item Mental Health Inventory (MHI; [47]). The MHI measures positive affect (e.g., “Have you felt calm and peaceful?”), anxiety (e.g., “Have you felt tense or high-strung?”), depression (e.g., “Have you been in low or very low spirits?”), and behavioural control (e.g., “Have you felt emotionally stable?”) over the 4 weeks prior to administration. Participants scored items on a six-point scale ranging from none of the time (1) to all of the time (6). A higher score indicates more psychological distress. Cronbach’s alpha was .94.

Quality-of-lifewas assessed using the World Health Organization Quality-of-Life BREF-questionnaire (WHO QOL-BREF), which is an abbreviated 26-item version of the WHO QOL-100 [42, 48]. It measures quality-of-life experienced by participants the last 4 weeks prior to administration. The WHO QOL-BREF contains one item from each of the 24 facets of quality-of-life included in the WHO QOL-100 (e.g., “How satisfied are you with the support you get from your friends?”, “How satisfied are you with yourself?”), plus two items on the overall quality-of-life and general health. Items were rated on a five-point scale ranging from none of the time (1) to all of the time (5). A higher score is indicative of greater quality-of-life. Cronbach’s alpha was .94.

Coping was assessed using the 28-item brief Coping Orientation to Problem Experience Scale (brief COPE scale; [5]), which is a brief form of the COPE-inventory [6]. The 28 item-scale comprises two 14-item subscales measuring maladaptive and adaptive coping. At the same time, it comprises two items for each of the 14 coping strategies. Items were rated on a four-point scale ranging from I have not been doing this at all (1) to I have been doing this a lot (4) with higher scores indicating more use of that particular coping strategy. The Cronbach’s alphas for each of the 14 coping strategies are presented in Table 2.

**Table 2:** Reliability coefficients of applied coping strategies

Coping strategy	Cronbach’s alpha
Adaptive coping	.82
Active coping	.62
Seeking emotional support	.77
Seeking instrumental support	.78
Planning	.60
Positive reframing	.75
Acceptance	.67
Humour	.68
Maladaptive coping	.76
Self-distraction	.71
Denial	.74
Substance use	.90
Behavioural disengagement	.58
Turning to religion	.86
Venting	.72
Self-blame	.87

Demographic and background variables, such as age, gender, and educational attainment, were also assessed.

### Mediation analyses

The present study explored the effects of seven adaptive and seven maladaptive coping strategies on the associations between SBA and family burden, on the one hand, and psychological distress and quality-of-life, on the other hand. To explore the mediational effects on these relationships, various mediation methods can be used [27, 36]. We opted for the bootstrapping method [34], which is a non-parametric test, and as such, does not violate assumptions of normality. It also increases statistical power and can be conducted with multiple simultaneous mediators to both determine if an overall effect exists and determine the effect of each of the mediators [35].

**Fig. 1:**
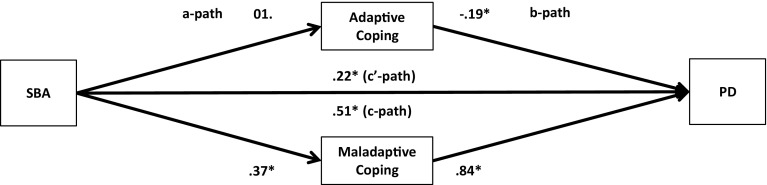
Unstandardized regression coefficients for the relationship between stigma by association (SBA) and psychological distress (PD) as mediated by adaptive and maladaptive coping. *p < .05

**Fig. 2:**
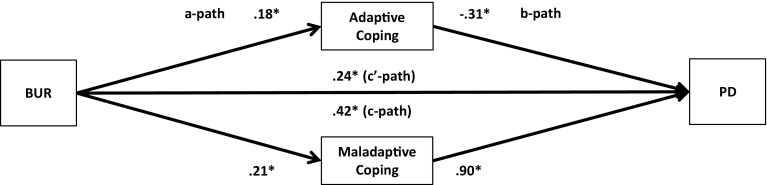
Unstandardized regression coefficients for the relationship between family burden (BUR) and psychological distress (PD) as mediated by adaptive and maladaptive coping. *p < .05

**Fig. 3:**
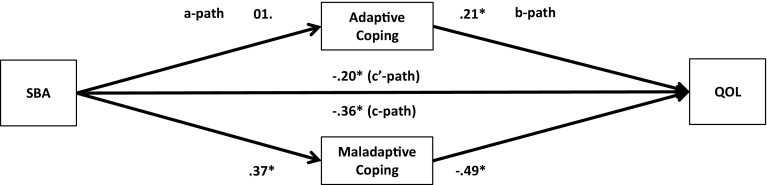
Unstandardized regression coefficients for the relationship between stigma by association (SBA) and quality-of-life (QOL) as mediated by adaptive and maladaptive coping. *p < .05

**Fig. 4:**
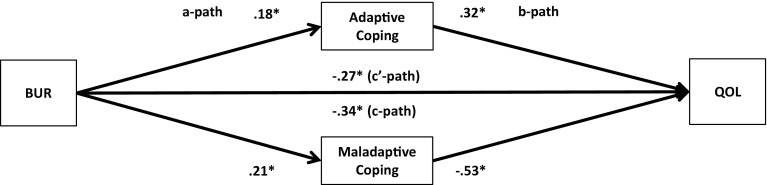
Unstandardized regression coefficients for the relationship between family burden (BUR) and quality-of-life (QOL) as mediated by adaptive and maladaptive coping. *p < .05

**Fig. 5:**
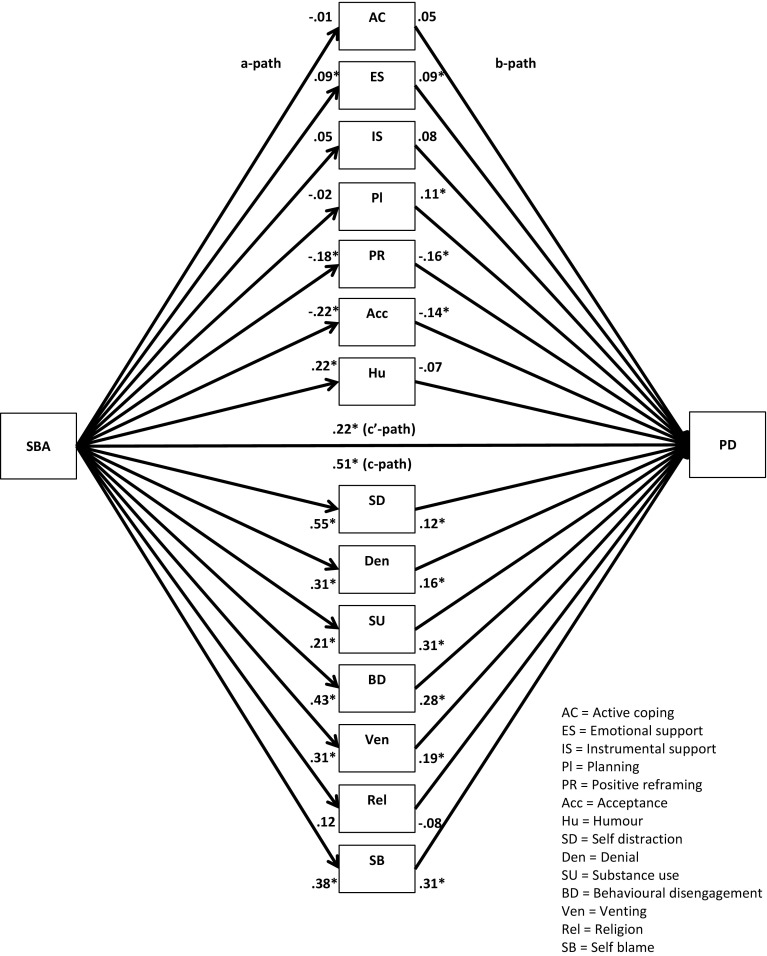
Unstandardized regression coefficients for the relationship between stigma by association (SBA) and psychological distress (PD) as mediated by 14 coping strategies. *p < .05

**Fig. 6:**
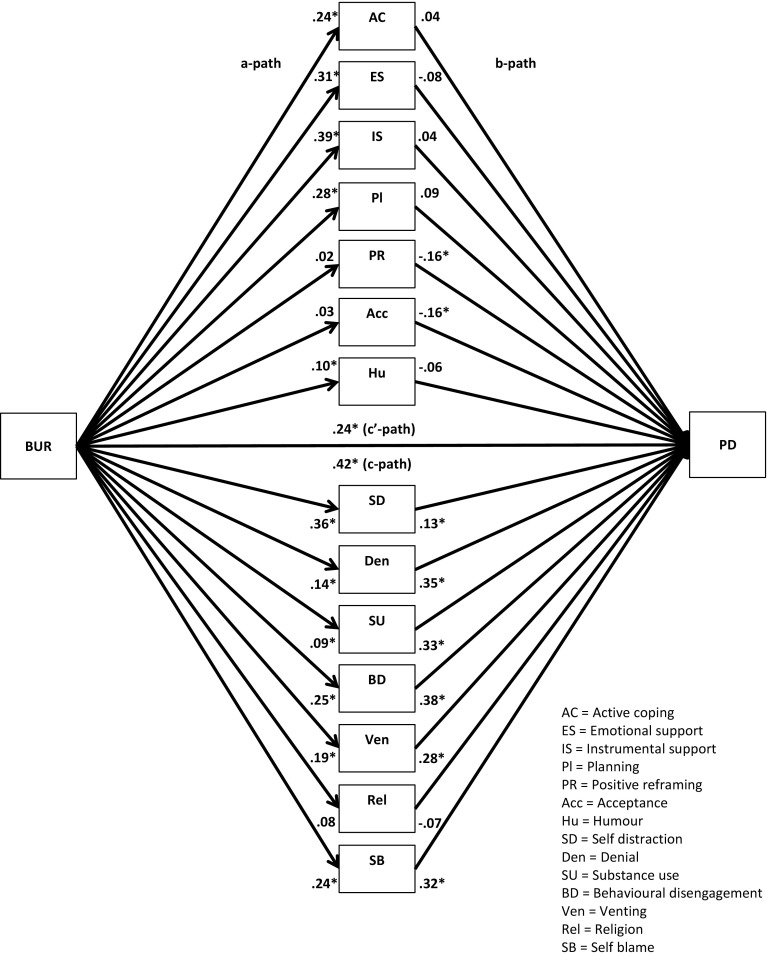
Unstandardized regression coefficients for the relationship between family burden (BUR) and psychological distress (PD) as mediated by 14 coping strategies. *p < .05

**Fig. 7:**
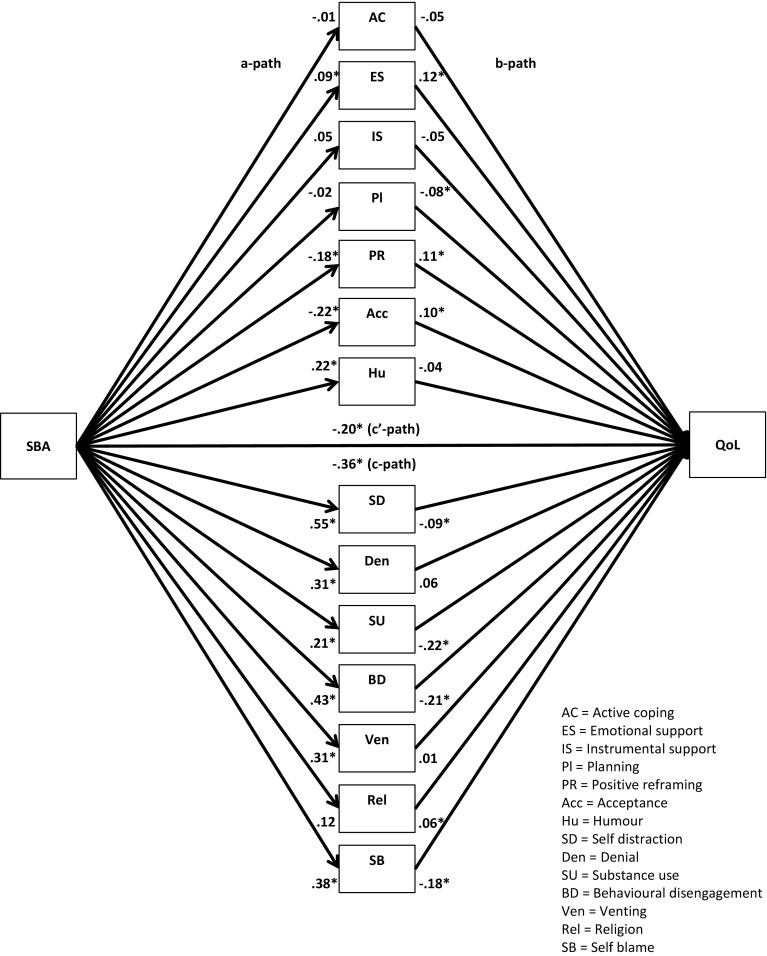
Unstandardized regression coefficients for the relationship between stigma by association (SBA) and quality-of-life (QOL) as mediated by 14 coping strategies. *p < .05

**Fig. 8:**
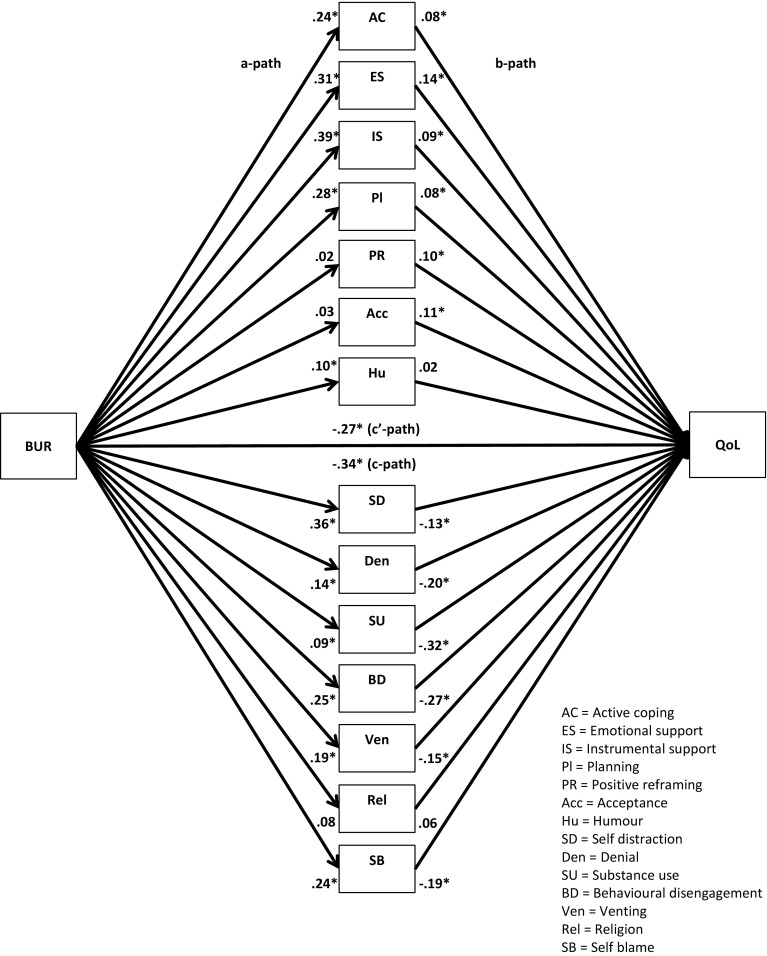
Unstandardized regression coefficients for the relationship between family burden (BUR) and quality-of-life (QOL) as mediated by 14 coping strategies. *p < .05

Descriptive statistics, correlational analyses, and multiple mediator analyses with a 95 % bias-corrected bootstrap confidence interval were used to analyse the data [18, 34]. Following the procedures developed by Preacher and Hayes [34], we, in our analyses, not only investigated the total effect of the independent variables and the mediator variables on the dependent variables (*c*-path) and the direct effect of the independent variables on the dependent variables (*c*′-path), but also the relationships between the independent variables and the mediator variables (*a*-paths) and between the mediator variables and the dependent variables (*b*-paths) (Figs. 1, 2, 3, 4, 5, 6, 7, 8).

## Results

In total, 12 % of the participants reported having experienced SBA to a medium or high amount and 45 % reported experiencing family burden to a medium or high amount. SBA and family burden were both positively correlated with psychological distress and negatively correlated with quality-of-life. SBA was also positively related to maladaptive coping, while family burden was positively related to both adaptive and maladaptive coping. Adaptive coping, in turn, was positively related to quality-of-life, whereas maladaptive coping was positively related to psychological distress and negatively related to quality-of-life. These correlations, along with the means and standard deviations for the primary study variables, are presented in Table 3.

**Table 3:** Means, standard deviations, and intercorrelations for main study variables

Variable	M	SD	1	2	3	4	5	6
1. Stigma by association	1.66	.60	–	.40***	.39***	−.36***	.01	.58***
2. Family burden	2.58	.61		–	.32***	−.34***	.27***	.35***
3. Psychological distress	2.87	.78			–	−.79***	−.03	.48***
4. Quality-of-life	3.60	.61				–	.09*	−.38***
5. Adaptive coping	2.15	.41					–	.17***
6. Maladaptive coping	1.39	.37						–

* *p* < .05, ** *p* < .01, *** *p* < .001

Multiple regression analyses that simultaneously assessed the relationships between SBA and family burden, on the one hand, and psychological distress and quality-of-life on the other demonstrated that SBA and family burden explained 18.6 % of the variance in psychological distress [*R*^*2*^ = .19, *F*(3500) = 57.29, *p* < .001] and 17.3 % of the variance in quality-of-life [*R*^*2*^ = .17, *F*(3500) = 52.12, *p* < .001]. Both SBA and family burden predicted psychological distress (*β* = .311, *p* < .001 and β = .200, *p* < .001, respectively) and quality-of-life (*β* = −.266, *p* < .001 and *β* = −.230, *p* < .001, respectively). Because both SBA and family burden remained significant in these simultaneous analyses, it is evident that both account for a unique variance in psychological distress and quality-of-life.

To examine the mediating role of coping, we first explored the frequency with which participants used specific coping strategies. To do this, we calculated the average of the two items representing each of the 14 coping strategies. The results showing the percentage of participants that frequently (i.e., value ≥3.0) applied a specific coping strategy are presented in Table 4 and indicated that participants used adaptive coping strategies more often than maladaptive coping strategies.

**Table 4:** Frequencies of applied coping strategies (*N* = 503)

Coping strategy	Percentage (%)
Acceptance^a^	62.0
Positive reframing^a^	43.9
Planning^a^	34.8
Active coping^a^	25.0
Seeking instrumental support^a^	19.7
Seeking emotional support^a^	13.9
Turning to religion^b^	11.7
Self-distraction^b^	8.0
Self-blame^b^	4.7
Humour^a^	4.2
Substance use^b^	2.6
Venting^b^	2.6
Behavioural disengagement^b^	2.2
Denial^b^	1.0

^a^Adaptive coping style

^b^Maladaptive coping style

Next, multiple parallel mediation analyses were conducted [18, 34]. We first examined whether adaptive coping and maladaptive coping mediate the relationships between, on the one hand, SBA and family burden, and, on the other, psychological distress and quality-of-life. These results are displayed in Figs. 1, 2, 3, and 4, and Tables 5 and 6).

**Table 5:** Indirect effects of stigma by association, respectively, family burden on psychological distress through adaptive and maladaptive coping strategies

	Stigma by association BCa* 95 % CI			Family burden BCa* 95 % CI		
	Point estimate	Lower limit CI	Upper limit CI	Point estimate	Lower limit CI	Upper limit CI
Adaptive coping	−.0005	−.0314	.0142	−.0563	−.0993	−.0262
Active coping	−.0007	−.0136	.0069	.0093	−.0161	.0402
Emotional support	−.0057	−.0305	.0031	−.0248	−.0696	.0110
Instrumental support	.0042	−.0042	.0292	.0144	−.0310	.0629
Planning	−.0026	−.0225	.0085	.0248	−.0063	.0701
Positive reframing	.0313	.0070	.0716	−.0033	−.0283	.0155
Acceptance	.0326	.0092	.0707	−.0051	−.0294	.0128
Humour	−.0158	−.0502	.0065	−.0053	−.0256	.0032
Maladaptive coping	.3078	.2207	.4089	.1911	.1442	.2588
Self-distraction	.0640	.0077	.1234	.0465	.0108	.0866
Denial	.0529	.0034	.1197	.0481	.0236	.0872
Substance use	.0664	.0319	.1355	.0309	.0110	.0693
Behavioural disengagement	.0529	.0034	.1197	.0991	.0582	.1528
Venting	.0607	.0168	.1123	.0541	.0254	.0979
Religion	−.0091	−.0308	.0001	−.0055	−.0224	.0014
Self-blame	.1180	.0674	.1801	.0765	.0423	.1250

*BCa Bias-corrected and accelerated bootstrapping confidence intervals. Confidence Intervals containing zero are interpreted as not significant

**Table 6:** Indirect effects of stigma by association, respectively, family burden on quality-of-life through adaptive and maladaptive coping strategies

	Stigma by association BCa* 95 % CI			Family burden BCa* 95 % CI		
	Point estimate	Lower limit CI	Upper limit CI	Point estimate	Lower limit CI	Upper limit CI
Adaptive coping	.0005	−.0163	.0244	.0597	.0323	.0963
Active coping	.0006	−.0055	.0120	.0183	.0008	.0437
Emotional support	.0112	.0004	.0407	.0438	.0151	.0820
Instrumental support	−.0028	−.0239	.0031	.0339	.0041	.0682
Planning	.0018	−.0063	.0172	.0234	.0001	.0519
Positive reframing	−.0218	−.0517	−.0040	.0022	−.0104	.0202
Acceptance	−.0216	−.0516	−.0034	.0035	−.0083	.0214
Humour	.0079	−.0099	.0323	.0021	−.0052	.0154
Maladaptive coping	−.1808	−.2634	−.1153	−.1118	−.1744	−.0762
Self-distraction	−.0499	−.1036	−.0105	−.0498	−.0873	−.0196
Denial	.0188	−.0201	.0623	−.0282	−.0663	−.0103
Substance use	−.0468	−.0964	−.0211	−.0222	−.0513	−.0071
Behavioural disengagement	−.0924	−.1610	−.0470	−.0695	−.1136	−.0398
Venting	.0024	−.0364	.0407	−.0288	−.0583	−.0072
Religion	.0076	.0000	.0257	.0045	−.0012	.0173
Self-blame	−.0709	−.1149	−.0346	−.0456	−.0778	−.0228

*BCa* Bias-corrected and accelerated bootstrapping confidence intervals. Confidence Intervals containing zero are interpreted as not significant

The regression coefficient between SBA and adaptive coping (.01) was statistically not significant. The regression coefficients between adaptive coping and psychological distress (−.19) and between adaptive coping and quality-of-life (.21), however, were both statistically significant (Figs. 1, 3). We, subsequently, tested the significance of the indirect effects using the bootstrapping procedures. The bootstrapped unstandardized indirect effects were .0005 (i.e., on the relationship between SBA and psychological distress) and .0005 (i.e., on the relationship between SBA and quality-of-life). The 95 % confidence intervals for the relationship between SBA and psychological distress ranged from −.0314 (lower limit confidence interval) to .0142 (upper limit confidence interval) and from −.0163 to .0244 for the relationship between SBA and quality-of-life(Tables 5, 6). If zero falls within the lower limit and upper limit confidence interval, then we can conclude that the indirect effect for this mediator is not significant [18, 34]. Thus, the relationships between SBA and psychological distress and between SBA and quality-of-life were not significantly mediated by adaptive coping. The regression coefficient for the relationship between family burden and adaptive coping, however, was statistically significant (.18), as were the regression coefficients for the relationship between adaptive coping and psychological distress (−.31) and the relationship between adaptive coping and quality-of-life (.32) (Figs. 2, 4). We, subsequently, tested the significance of the indirect effects using the bootstrapping procedures. The bootstrapped unstandardized indirect effects were −.0563 for the relationship between family burden and psychological distress and .0597 for the relationship between family burden and quality-of-life. The confidence intervals ranged from −.0993 to −.0262 for the relationship between family burden and psychological distress and from .0323 to .0963 for the relationship between family burden and quality-of-life. Thus, the relationships between family burden and psychological distress and between family burden and quality-of-life were significantly mediated by adaptive coping.

Maladaptive coping was investigated using the same method and was a significant mediator on the relationships between SBA and psychological distress (indirect effect = .31), SBA and quality-of-life (−.18), family burden and psychological distress (.19), and family burden and quality-of-life (−.11) (Figs. 1, 2, 3, 4; Tables 5, 6).

We then examined the extent to which the 14 coping strategies mediated the relationships between SBA and family burden, on the one hand, and psychological distress and quality-of-life, on the other hand. These results are displayed in Figs. 5, 6, 7, and 8, and Tables 5 and 6. Significant mediators of the relationship between SBA and psychological distress were positive reframing (indirect effect = .03), acceptance (.03), self-distraction (.06), denial (.05), substance use (.07), behavioural disengagement (.05), venting (.06), and self-blame (.12). Significant mediators of the relationship between family burden and psychological distress were self-distraction (.05), denial (.05), substance use (.03), behavioural disengagement (.10), venting (.05), and self-blame (.08). Significant mediators of the relationship between SBA and quality-of-life were emotional support (.01), positive reframing (−.02), acceptance (−.02), self-distraction (−.05), substance use (−.05), behavioural disengagement (−.10), and self-blame (−.07). Significant mediators of the relationship between family burden and quality-of-life were active coping (.02), emotional support (.04), instrumental support (.03), planning (.02), self-distraction (−.05), denial (−.02), substance use (−.02), behavioural disengagement (−.07), venting (−.05), and self-blame (−.05).

In these analyses, we examined ‘gender’, ‘educational attainment’, ‘familial relation’, and ‘type of mental illness’ as covariates and found no effects for gender, familial relational, and type of mental illness. Educational attainment, however, was a significant covariate. For this reason, we controlled for educational attainment in all mediation analyses [34].

## Discussion

This study is among the first to examine the relationships between SBA, family burden, psychological distress, quality-of-life, and coping among family members of PWMI. The effect sizes for the relationships between SBA and family burden, on the one hand, and psychological distress, respectively, quality-of-life on the other hand, were both just under 20 %, and as such, SBA and family burden appear to negatively affect the well-being of a considerable group of people. However, we may, consequently, presume that there are other important variables (e.g., employment status, physical activity, vulnerability to stigma, or chronic conditions) that are also associated with psychological distress and quality-of-life [16, 39]. Our results further show that from a quantitative point of view, family burden seems to be a more substantial stressor for family members than SBA. It is possible that family burden is experienced more often than SBA due to its more direct, overt, and practical character [12, 24, 41].

Of greater importance, our research sheds light on both the prevalence and impact of coping strategies that family members employ. We found that participants more frequently applied adaptive coping than maladaptive coping. This is in line with Moore et al. [30] who found that family members endorsed using adaptive coping more often than maladaptive coping. The findings further showed that maladaptive coping strategies generally mediated the relationships between SBA and psychological distress, respectively, quality-of-life and between family burden and psychological distress, respectively, quality-of-life, whereas adaptive coping strategies mainly mediated the relationships between family burden and psychological distress, respectively, quality-of-life. This may indicate that there are other important variables that are also associated with psychological distress and quality-of-life.

These findings suggest that family members are likely to take action or engage in adaptive coping strategies when their well-being is threatened by family burden and, to a lesser extent, SBA. As such, family members could be described as seeking external solutions for what are perceived to be external problems. It is also possible that family members felt that they had more resources and capabilities to cope with family burden than with SBA [25, 28]. Because family burden has a more direct, overt, and practical nature than SBA [46], family burden might be perceived as more changeable, controllable, or manageable than SBA, and this might evoke more adaptive coping. Adaptive coping strategies likely target and alter the perceived stressor directly and thus improve one’s personal situation. These adaptive coping strategies may also make family members more aware of possibilities to actively improve their personal and familial situation, thus reflecting overall life satisfaction. Furthermore, SBA appeared to diminish the use of positive reframing and acceptance as coping strategies (*a*-paths). Nonetheless, positive reframing and acceptance were still associated with decreased psychological distress and increased quality-of-life (*b*-paths). They may, therefore, make the source of stress seem less negative and could be a precursor to providing social support to this family member, which, in turn, might improve his or her condition. This is in line with Carver et al. [6], who suggested that framing a stressor as something positive can intrinsically lead to a continuation or resumption of adaptive coping actions.

Maladaptive coping strategies emerged as significant mediators when examining the relationships between SBA and family burden, on the one hand, and psychological distress and quality-of-life, on the other hand. Family members who perceive SBA may use maladaptive coping strategies to escape or avoid feelings of distress, which eventually can lead to inactivity, apathy, fantasy, detachment, or feelings of hopelessness [1, 30, 31]. The use of maladaptive coping strategies thus appears to be a means by which family members attempt to distance themselves from stigmatising situations or their family member with mental illness. Maladaptive coping strategies also appear to, at least some of the time, reduce the intensity with which negative emotions resulting from SBA and family burden are felt. However, maladaptive coping strategies do not seem to actually alter the stigmatized condition and its negative outcomes. We can thus presume that, in the long run, negative emotions are likely to reappear and increase psychological distress while decreasing quality-of-life. These findings are consistent with findings put forth by Fortune et al. [15] who, in their study among relatives of patients with schizophrenia, found that seeking emotional support and active coping were associated with less psychological distress, while coping through self-blame was related to increased psychological distress.

Finally, our findings suggest that higher educational attainment is related to diminished distress. This is in line with Brännlund and Hammarström [3] who found that higher educational attainment is linked to diminished psychological distress, which can potentially be understood in light of the mechanisms of social and labour-market resources.

In sum, the findings suggest that family members experience psychological distress and lower quality-of-life when they share the stigma and the practical issues that arise from having a family member with mental illness, and that they use various adaptive and maladaptive coping strategies to mitigate the negative outcomes of SBA and family burden. Adaptive coping strategies are used more often than maladaptive coping strategies and this is positive, because most adaptive coping strategies mitigate the negative outcomes of family burden, and to a lesser extent of SBA, while maladaptive coping strategies increase psychological distress and decrease quality-of-life in the context of both SBA and family burden.

### Study strengths and limitations

The relative novelty of research on coping with SBA and family burden among family members of PWMI is the primary strength of this study. The large number of participants is another strength, as is the broad range of mental illnesses involved. A final strength is the use of advanced multiple mediation analyses and the bootstrapping techniques. Although various mediation methods can be used to explore mediational effects, the mediation bootstrapping method we used is a non-parametric test and, as such, does not violate assumptions of normality. It also increases statistical power [34]. However, the generalisability of our results may be limited by, first, the fact that our study was cross section, which disallows for causal conclusions, and second, we used self-reports that could possibly lead to response bias [10]. Finally, our findings could be limited by our methods. We assumed that the mediation analysis model reflects the correct underlying model and processes and that no important variables were omitted from the model [29]. As the effect sizes of SBA and family burden, however, were both just under 20 %, we can presume that there are other important variables that are also associated with psychological distress and quality-of-life [16]. These variables should be the subject of further research.

### Theoretical and practical implications

The findings of our study have implications for both practice and theory. Given the relative novelty of research on coping with SBA and family burden among family members of PWMI, and especially, given our detailed exploration of the impact of 14 coping strategies, the present study contributes considerably to the literature on SBA and family burden. Most fundamentally, the findings show that the stigma of mental illness not only harms PWMI but also their immediate family members. Moreover, because adaptive coping strategies are much more helpful than maladaptive coping strategies, information on the relative effectiveness of these coping strategies should be actively promoted. Support in the use of adaptive coping strategies should be made readily available to family members. In this context, mental health care professionals can play an important role in helping family members to develop and apply advantageous coping skills [33].

## Conclusion

This study set out to provide additional insight with regard to SBA and family burden among family members of PWMI, and showed that SBA and family burden increase psychological distress and diminish quality-of-life among family members. It also demonstrated that several adaptive coping strategies mitigate the negative impact of SBA and family burden, whereas most maladaptive coping strategies increase the negative impact of SBA and family burden. We recommend that future research explore the experiences and consequences of SBA longitudinally, and, in doing so, investigate the effect of adaptive and maladaptive coping strategies on the relationships between SBA, psychological distress, and quality-of-life, in both the short term and in the long run. In addition, we recommend that more research on psychological distress and quality-of-life among family members be conducted to identify other important variables that may play a role in the well-being of family members.
